# Yttrium-90 radioembolization as an in vivo immune modulator: clinical evidence and pharmacologic implications

**DOI:** 10.3389/fphar.2026.1770097

**Published:** 2026-02-11

**Authors:** Shiwei Tang, Jia Cai, Pandi Wu, Guanwu Wang

**Affiliations:** Department of Interventional Radiology, Weifang People’s Hospital, Shandong Second Medical University, Weifang, Shandong, China

**Keywords:** hepatocellular carcinoma, immune modulation, immunotherapy combination, tumor immunity, yttrium-90 radioembolization

## Abstract

Yttrium-90 (Y-90) transarterial radioembolization (TARE) is widely used for the treatment of primary and metastatic liver tumors and has traditionally been viewed as a purely locoregional radiotherapeutic modality. However, accumulating clinical evidence supports the concept that Y-90 TARE can induce measurable immunologic changes in patients, detectable both systemically and within the tumor microenvironment. Longitudinal analyses of peripheral blood and tumor tissue from patients with hepatocellular carcinoma and liver metastases demonstrate transient immune activation, myeloid remodeling, and adaptive immune perturbations following treatment. Notably, these immune-stimulatory signals may coexist with counter-regulatory mechanisms, including upregulation of immune checkpoint pathways, suggesting a dynamic balance between immune priming and adaptive resistance. In this Perspective, we synthesize available *in vivo* human evidence supporting the concept that Y-90 TARE functions as an immune modulator rather than a solely cytotoxic intervention. We discuss the pharmacologic implications of these findings, particularly in relation to treatment sequencing, biomarker development, and rational combination strategies with immunotherapies. Recognizing Y-90 TARE as an immunologically active modality may inform the design of future clinical trials and optimize its integration into combination regimens aimed at durable tumor control.

## 1 Introduction

Radiation therapy has long been recognized to exert immunomodulatory effects beyond direct tumor cell killing, including antigen release, inflammatory signaling, and modulation of the tumor microenvironment. In this context, Yttrium-90 (Y-90) transarterial radioembolization (TARE) represents a distinctive form of internal radiation therapy, delivering high-dose beta radiation selectively to liver tumors via intra-arterial microspheres. Despite its extensive clinical use in hepatocellular carcinoma (HCC) and liver-dominant metastatic disease, Y-90 TARE has historically been conceptualized as a locoregional cytotoxic therapy with minimal systemic immunologic consequences (Ruohoniemi et al., 2020; Salem et al., 2013).

This view is increasingly challenged by emerging clinical data. Multiple studies employing longitudinal immune profiling in treated patients now suggest that Y-90 TARE induces reproducible changes in circulating immune cell populations, inflammatory mediators, and tumor-associated immune programs (Yu et al., 2024; Lee et al., 2023; Malone et al., 2025). A seminal Gut study demonstrated that sustained clinical responses to Y-90 TARE in HCC are associated with enhanced adaptive immune activation and increased effector cell signatures in both tumor tissue and peripheral blood, directly linking immune engagement with clinical outcomes (Chew et al., 2019). Furthermore, a related Gut commentary elaborated on this study’s own data, emphasizing that Y-90-induced immune responses call for early application of multiple immune checkpoint blockers to overcome emerging adaptive resistance and maximize clinical benefit (Rivoltini et al., 2023). These observations raise an important question with direct pharmacologic relevance: should Y-90 TARE be considered an *in vivo* immune modulator, and if so, how can this property be therapeutically exploited?

In this Perspective, we argue that sufficient human *in vivo* evidence exists to support the classification of Y-90 TARE as an immunologically active intervention. We synthesize key findings from clinical studies, discuss the dual nature of immune activation and suppression following treatment, and outline implications for combination strategies and biomarker-guided therapy.

## 2 Scope and evidence selection

This Perspective is based on a focused narrative synthesis of published human clinical studies evaluating immunologic effects of Y-90 TARE. Relevant literature was identified through targeted searches of PubMed and Google Scholar up to March 2025, using combinations of the terms “Yttrium-90,” “radioembolization,” “immune,” “immunomodulation,” “immunotherapy,” “checkpoint inhibitor,” and “hepatocellular carcinoma.”

Priority was given to prospective trials, translational clinical studies incorporating immune profiling, and high-quality retrospective analyses reporting systemic or intratumoral immune endpoints. Studies limited to dosimetry, technical optimization, or purely preclinical models without patient immune correlates were not included. This article does not aim to provide a systematic or exhaustive review but rather to synthesize representative *in vivo* human evidence relevant to the question of whether Y-90 TARE functions as an immune-modulating intervention with pharmacologic implications. We acknowledge that heterogeneity among the included studies, including differences in patient populations, treatment settings, immune assays, and sampling timepoints, may influence the observed immune signals. In addition, the possibility of publication bias toward studies reporting positive immunologic findings cannot be fully excluded. These limitations should be considered when interpreting the conclusions of this Perspective.

## 3 Defining immune modulation in the clinical setting

For the purposes of this discussion, immune modulation refers to treatment-induced changes in immune state that are measurable in patients and plausibly relevant to antitumor immunity. In the context of Y-90 TARE, these endpoints include: systemic immune changes, such as shifts in circulating lymphoid and myeloid populations, cytokine profiles, and activation markers; local immune remodeling, encompassing alterations in tumor-infiltrating immune cells, immune gene expression signatures, and checkpoint pathway activation within the liver or tumor tissue; and temporal dynamics, including the onset, magnitude, and duration of immune perturbations following treatment.

Importantly, immune modulation does not imply durable immune memory or clinical benefit *per se*. Rather, in the context of this Perspective, immune modulation refers to measurable and often transient immune changes observed in patients following Y-90 TARE, and should not be interpreted as definitive evidence of immune-mediated clinical efficacy.

## 4 Human in vivo evidence of immune modulation after Y-90 TARE

### 4.1 Systemic immune perturbations

**FIGURE 1:**
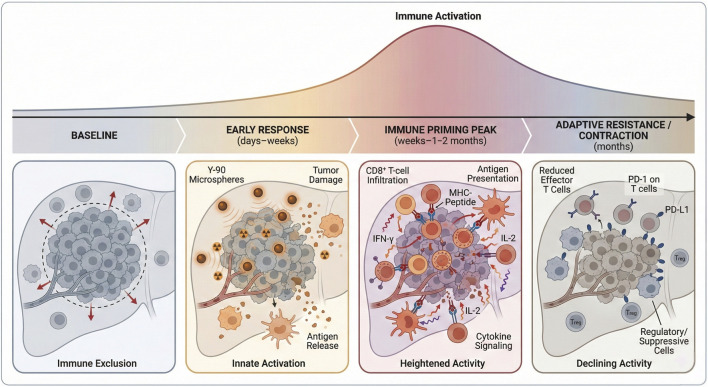
Temporal immune trajectory following Yttrium-90 radioembolization. Schematic illustration of the dynamic immune changes observed after Yttrium-90 (Y-90) transarterial radioembolization (TARE) in liver tumors. At baseline, tumors exhibit low immunogenicity and limited immune cell infiltration. In the early post-treatment phase (days to weeks), radiation-induced tumor injury leads to antigen release and activation of innate immune pathways. This is followed by a transient peak phase (weeks to ∼1–2 months) characterized by enhanced antigen presentation, increased CD8^+^ T-cell infiltration, and systemic immune modulation. Over subsequent months, immune activity declines and adaptive resistance mechanisms, including immune checkpoint upregulation, emerge, resulting in immune contraction. The figure highlights the time-dependent nature of immune modulation after Y-90 TARE and its implications for combination strategies with immunotherapy. Figure generated with Biorender.

Several clinical studies have conducted longitudinal immune monitoring in patients undergoing Y-90 TARE, particularly in HCC (Malone et al., 2025; Kaya et al., 2023). These investigations consistently report changes in circulating immune cell subsets within weeks of treatment. Observed effects include transient activation of T-cell populations, shifts in effector-to-regulatory ratios, and alterations in myeloid cell phenotypes. In parallel, changes in circulating cytokines and inflammatory mediators suggest engagement of innate immune pathways (Figure 1), which illustrates the temporal window of immune perturbation following Y-90 TARE and its potential relevance for treatment sequencing and combination timing with immunotherapy.

Prospective clinical data in breast cancer liver metastases further support immune engagement after radioembolization, with immune activation features associated with response assessed alongside PET/CT (Deipolyi et al., 2023).

A notable feature across studies is the temporal nature of these effects. Immune activation typically peaks within the first one to 2 months following TARE and diminishes thereafter, consistent with a window of immune perturbation rather than sustained systemic inflammation (Yu et al., 2024; Lee et al., 2023; Kaya et al., 2023). This pattern supports the concept of Y-90 TARE as a short-acting immune stimulus rather than a chronic immune driver.

### 4.2 Tumor and liver microenvironment remodeling

Evidence for local immune modulation has emerged from analyses of tumor tissue and liver biopsies obtained before and after Y-90 TARE. Transcriptomic and immunohistochemical studies reveal changes in immune-related gene expression programs, including pathways associated with antigen processing, interferon signaling, and immune cell recruitment (Malone et al., 2025; Kaya et al., 2023). Importantly, these changes have been linked to radiographic response categories, suggesting biological relevance rather than nonspecific tissue injury.

In metastatic colorectal cancer, staged lobar treatment designs have provided a unique opportunity to assess immune effects in untreated contralateral liver tumors. Such studies demonstrate immune alterations beyond the directly irradiated field, implying that Y-90 TARE can elicit regional or systemic immunologic signaling rather than purely local effects (Kaya et al., 2023; Fabritius and Ricke, 2022).

## 5 Immune priming and adaptive resistance: a dual-edged response

While immune activation following Y-90 TARE is increasingly well documented, it is equally important to recognize accompanying counter-regulatory mechanisms. Several studies report increased expression of immune checkpoint molecules, including PD-1 on T cells and PD-L1 on myeloid or tumor cells, following treatment (Yu et al., 2024; Lee et al., 2023; Kaya et al., 2023). These findings suggest that immune priming may be rapidly tempered by adaptive resistance pathways.

This dual response mirrors observations made with other radiotherapeutic and locoregional interventions, where inflammatory signaling and antigen release coexist with immunosuppressive feedback loops. From a pharmacologic perspective, this balance underscores why immune activation alone may be insufficient for durable tumor control and why rational combination strategies are required.

## 6 Limitations and conflicting clinical evidence

Despite growing interest in the immunologic consequences of Y-90 TARE, it is important to acknowledge that immune activation following treatment is heterogeneous and not uniformly associated with clinical benefit. Large randomized trials comparing Y-90 radioembolization with sorafenib in advanced hepatocellular carcinoma, including the SARAH and SIRveNIB studies, failed to demonstrate an overall survival advantage for Y-90 in unselected patient populations, underscoring that biologic activity does not necessarily translate into improved outcomes (Vilgrain et al., 2017; Chow et al., 2018).

Moreover, immune perturbations observed after TARE are often transient and may reflect a combination of tumor-directed immune priming and nonspecific hepatic inflammation or lymphocyte depletion. Prospective immune monitoring studies have reported variable trajectories, including delayed lymphopenia, raising the possibility that post-TARE immune landscapes may in some cases constrain rather than enhance responsiveness to immunotherapy (Kitsel et al., 2024). Together, these findings emphasize that immune modulation by Y-90 TARE should be viewed as context-dependent and hypothesis-generating, rather than intrinsically predictive of therapeutic synergy.

## 7 Pharmacologic implications and combination strategies

### 7.1 Treatment sequencing and timing

The transient nature of immune perturbations after Y-90 TARE highlights the importance of timing (Malone et al., 2025). Immunotherapies administered too early may fail to capitalize on antigen release, whereas delayed treatment may miss the window of immune priming altogether. Prospective studies incorporating defined post-TARE sampling timepoints are needed to identify optimal sequencing strategies.

### 7.2 Beyond PD-1/PD-L1 blockade

While early clinical efforts have focused on combining Y-90 TARE with PD-1/PD-L1 inhibitors, the observed immune landscape suggests additional opportunities (Yu et al., 2024; Kaya et al., 2023; Cannella et al., 2022). Modulation of myeloid populations, targeting of immunosuppressive cytokine pathways, or engagement of innate immune sensors may further enhance the immunogenic potential of TARE. These strategies remain largely hypothetical but are grounded in observed immune alterations in treated patients.

### 7.3 Biomarker development

Recognition of Y-90 TARE as an immune modulator necessitates biomarker development. Minimal immune monitoring panels incorporating circulating immune subsets, checkpoint expression, and inflammatory mediators could stratify patients most likely to benefit from combination approaches. Importantly, biomarkers must distinguish tumor-relevant immune activation from nonspecific hepatic inflammation (Malone et al., 2025; Kaya et al., 2023).

### 7.4 Safety considerations in TARE-immunotherapy combinations

While combining Y-90 TARE with immune checkpoint inhibitors is biologically appealing, emerging clinical data indicate that safety considerations are critical. Reported adverse events include immune-related hepatitis, hepatic decompensation, biliary injury, and radioembolization-induced liver disease, particularly in patients with limited hepatic reserve or underlying cirrhosis (Ruohoniemi et al., 2020; Gil-Alzugaray et al., 2013). Early retrospective and phase I/II studies suggest that TARE-ICI combinations are generally feasible, but rates of grade ≥3 hepatic toxicities appear higher in certain cohorts compared with TARE alone (Yu et al., 2024).

Importantly, attribution of liver injury in this setting is challenging, as radiation-induced damage, tumor progression, and immune-mediated inflammation may coexist (Malone et al., 2025). These uncertainties highlight the need for cautious patient selection, rigorous liver function monitoring, and prospective trial designs incorporating predefined safety endpoints. From a pharmacologic perspective, safety constraints may ultimately shape optimal sequencing and combination strategies as much as immunologic rationale.

## 8 Future directions

To advance the field, future studies should integrate immune monitoring as a core component rather than an exploratory endpoint. Harmonized sampling schedules, paired tissue analyses, and standardized immune assays will be essential. Importantly, immune findings should be correlated with clinical outcomes to establish predictive rather than merely descriptive biomarkers.

## 9 Conclusion

Accumulating human *in vivo* evidence suggests that Y-90 TARE induces measurable and biologically meaningful immune modulation in patients with liver tumors. These effects are characterized by transient immune activation accompanied by adaptive resistance mechanisms. Recognizing Y-90 TARE as an immunologically active intervention reframes its role within modern oncologic pharmacology and provides a rationale for biomarker-guided combination strategies. As clinical development continues, integrating immunologic endpoints into Y-90-based trials will be critical to fully harness its therapeutic potential.

## Data Availability

The original contributions presented in the study are included in the article/supplementary material, further inquiries can be directed to the corresponding author.
